# Brazilian Consumers’ Perception towards Food Labeling Models Accompanying Self-Service Foods

**DOI:** 10.3390/foods11060838

**Published:** 2022-03-15

**Authors:** Nariéli Felipetto, Patrícia Arruda Scheffer, Karen Mello de Mattos Margutti, Joice Trindade Silveira, Clandio Timm Marques, Cátia Regina Storck, Viviani Ruffo de Oliveira, Elizabete Helbig, Verônica Cortez Ginani, Ana Lúcia de Freitas Saccol

**Affiliations:** 1 Master’s Program in Sciences of Health and Life, Research Group in Food and Nutrition Security (GESAN/CNPq), Franciscan University (UFN), Santa Maria 97010-491, Brazil; narieli.felipetto@ufn.edu.br (N.F.); patricia.scheffer@ufn.edu.br (P.A.S.); 2 Nutrition Course, Life Sciences Knowledge Area, University of Caxias do Sul (UCS), Caxias do Sul 95070-560, Brazil; kmmmargutti@ucs.br; 3 Nutrition Course, Federal University of Pampa, Itaqui 97650-000, Brazil; joicesilveira@unipampa.edu.br; 4 Mathematics Course and Postgraduate Program in Science Teaching and Math, Franciscana University (UFN), Santa Maria 97010-000, Brazil; clandio@ufn.edu.br; 5 Nutrition Course, Research Group in Food and Nutrition Security (GESAN/CNPq), Franciscana University (UFN), Santa Maria 97010-491, Brazil; catiars@ufn.edu.br; 6 Department of Nutrition, Faculty of Medicine, Federal University of Rio Grande do Sul (UFRGS), Porto Alegre 90035-003, Brazil; vivianiruffo@hotmail.com; 7 Faculty of Nutrition, Federal University of Pelotas (UFPel), Pelotas 96010-900, Brazil; elizabete.helbig@ufpel.edu.br; 8 Department of Nutrition, College of Health Sciences, University of Brasilia (UnB), Brasilia 70910-900, Brazil; vcginani@gmail.com; 9 Postgraduate Program in Life Sciences, Research Group in Food and Nutrition Security (GESAN/CNPq), Franciscana University (UFN), Santa Maria 97010-491, Brazil

**Keywords:** consumer food behavior, healthy diet, food label, food hypersensitivity, Brazil

## Abstract

The study aimed to evaluate consumers’ perception of self-service foods’ nutrition labels. This qualitative and quantitative assessment was performed with potential consumers at food services. Four food labeling formats, traditional, simplified, traffic-light, and warning, were proposed to evaluate three types of sandwiches: simple, chicken, and hamburger. Data were collected via an online survey from April to May 2020. The study included 413 subjects. The respondents preferred the traffic-light format, but there was a good understanding and acceptability of all four models. The traffic-light and warning nutrition labeling models, which showed health warnings, led to a reduction in the choice of the Simple Sandwich and the Hamburger. Most respondents (96.1%, *n* = 397) agreed that it is necessary to complement the information on food labels with ingredients and the number of calories per serving. Therefore, it is essential to have legislation regulating such issues. Consumers’ choices improved with the increase in the information placed on the products. This research demonstrated that nutrition labels explain what exists currently and that consumers require such information. Thus, food labeling may positively influence consumers’ choices.

## 1. Introduction

Cardiovascular diseases, cancers, diabetes, and other non-communicable diseases (NCDs) were responsible for 71% (41 million) of the 57 million deaths globally in 2016. Furthermore, without distinction among population groups, NCDs are also a cause of death in younger populations, corresponding to 75% of premature deaths in adults (occurring in the age group 30 to 69 years). In Brazil, the data follow the rest of the world. Most deaths occur due to NCDs (70%). Among the NCDs, obesity has increased by 67.8% in the last thirteen years, from 11.8% in 2006 to 19.8% in 2018 [1]. In 2017, Brazil was the country with the fourth highest number of people with diabetes (20 to 79 years), reaching about 12.5 million people (11.4%) [2]. The hypertension prevalence ranged from 23 to 25% in the population over 18 years of age in recent years [3,4].

Considering the continental dimension of the country and the great diversity of realities, many people can benefit from health promotion actions. In this sense, the Brazilian government developed the “Strategic Action Plan to Combat NCDs in Brazil”. The Plan is based on three action guidelines: (a) surveillance, information, evaluation, and monitoring; (b) health promotion; and (c) comprehensive care. Healthy eating cuts across the three guidelines. However, it is highlighted in the “health promotion” guideline. Among the actions, food labeling regulation is the focus of large investments [5,6].

As in Brazil, other countries adopt strategies capable of contributing to a healthy diet to mitigate NCDs. One of them is the dissemination of information through food labels. Jáuregui et al. [7], for example, in their study, agree that front-of-pack (FOP) nutrition labeling is an important strategy to combat diseases resulting from poor diet. FOP directs food choices and has been adopted in different countries. The idea is to expose important information to consumers to consider when purchasing food. In this way, consumers’ understanding of food and their preferences and experiences allow an assessment of the nutritional quality of the product. It, thus, enables the purchase of food to occur more consciously, generating possible health benefits.

Therefore, communication information, such as FOP and nutrition and health claims (NHCs), integrates factors that interfere with consumers’ perception of food healthiness. However, the way the conveyed information acts on the subjects varies. In Poland, for example, a study identified that the self-rated understanding about nutritional health is the only significant predictor of interest in food labels. Another study with the same population highlighted the lack of trust in the label as an essential factor. It revealed the great complexity that exists in the dissemination of guidelines through labels. The choice of the best label must be prudent to meet the diversity of consumers and contexts [8,9].

Research has assessed the predictors that influence label reading at home and while shopping [9]. It also evaluated the label use behaviors of different populations towards the nutritional information [9,10]. The results revealed that consumers who usually check the nutritional data stated on the label eat more fruits, vegetables, whole grains, and beverages with low-sugar content than those who do not have this habit. Furthermore, positive associations between the understanding of nutrition and the verification of the nutritional information label have been reported [11].

Following a global trend, the Brazilian government has invested in standards that guide and require food producers to inform consumers about the food market. In Brazil, as in other countries, the Brazilian Health Surveillance Agency (ANVISA) establishes that food packages must show a table of nutrition information [12,13,14,15]. However, the authors of the Preliminary Regulatory Impact Analysis Report on Nutrition Labeling, published by ANVISA, detected some issues [16].

One of the report’s highlights was verifying consumers’ difficulty in using the information present on the labels. Another aspect was the absence of food labels on items prepared and offered in food services, such as restaurants, cafeterias, and bakeries. In this case, we should consider that eating out of the home usually involves consuming foods with high fat, sugar, and sodium contents and high energy density. Moreover, it is associated with unsatisfactory vitamin, fiber, and calcium consumption. One of the reasons is the servings. They are typically large and include highly palatable foods, which have contributed to excessive weight gain and, consequently, increases in NCDs [17,18,19].

Some solutions are described in the report mentioned above to improve consumer understanding of the information conveyed by food labels. The authors observed the tendency to adopt semi-interpretive nutrition labeling models, such as traffic-lights and warnings. This type of labeling proves to be more effective and focuses on qualifying the content of nutrients that impact food and health. After all, nutrition information serves to inform and clarify the nutritional quality of the food item available and not to confuse the consumer. Food labels must prompt consumers to make well-informed food choices that better align with their health interests [16].

Regarding the absence of information on the composition of products at food services, the ANVISA report proposes co-regulation as a possible method to address this regulatory issue [16]. The proposal considers the previous experience lived by ANVISA in 2010. At this time, the Public Federal Ministry and fast-food chain members of the National Restaurant Association and the Brazilian Franchising Association signed a Conduct Adjustment Term. The results revealed that the nutrition information panel displayed in food services has high technical complexity. Firstly, it requires managers, owners, and food handlers to standardize the preparation using technical preparation files (TPF). Furthermore, the ingredients must be standardized, expanding the partnership between the supplier and the establishment, among other managerial aspects. Different food services still lack nutrition expertise, time, cost, nutrition information for exotic ingredients, and the ability to provide accurate nutrition information. Other aspects are libel risk, customer dissatisfaction, limited space on the menu, menu variations, loss of flexibility in changing the menu, staff training, and employees’ resistance to change current practices [20]. Despite being ordinarily experienced by the food industry, it can be a tremendous challenge for food services in general [12,13].

However, the availability of nutritional information about the food results in many benefits related to the prevalence of NCDs. By providing nutritional information about a product, consumers will be aware of the possible benefits or undesirable effects caused by certain foods. Likewise, food service managers and owners will become conscious of the importance of respecting the right of consumers to have access to correct and precise information that will guide them in their purchasing decisions [21,22].

Nutritional information promotes stimulation in consumers and, consequently, alters psychological and physiological perceptions. Perception occurs when the consumer becomes aware of the sensation in front of the visual stimulus, involving filtering, interpretation, and the reconstruction of information [23,24]. For this reason, we should evaluate the consumer’s perceptions and preferences regarding how information is transmitted. Investigations are essential to guide public policies on the subject. Considering the above and the scarcity of studies that address food labels in different types of food services in Brazil, this work aimed to evaluate the perception of subjects in relation to the nutritional labels of foods offered in food services.

**Figure 1:**
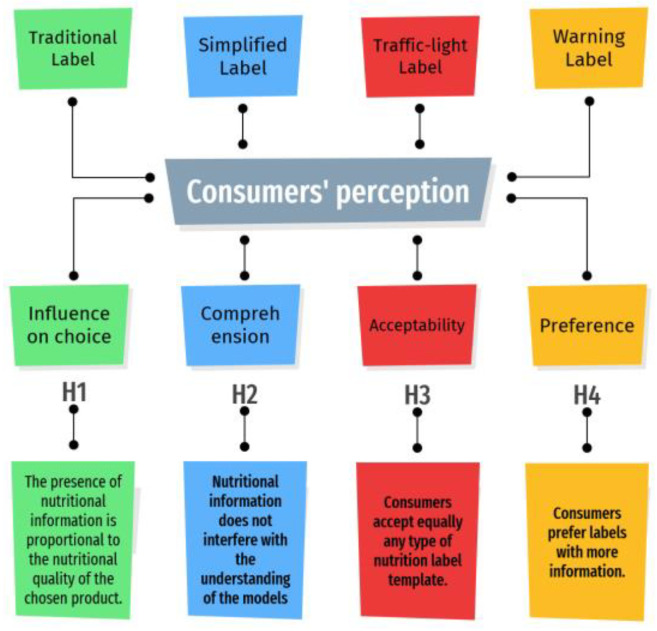
Hypotheses applied to assess the perception of subjects in relation to the nutritional labels of foods offered in food services. Legend: H, Hypotheses.

For the construction of this study, we elaborated the hypothesis presented in Figure 1.

The hypotheses presented (Figure 1) were based, at first, on studies on the influence of nutritional information on consumers’ food choices [25,26,27,28]. However, they also evaluated the possibility of the models not interfering with the understanding of the present content [8,25]. The central hypothesis was that more information would result in better choices and would be the consumer-preferred model [26,27].

## 2. Materials and Methods

### 2.1. Survey Subjects

This qualitative and quantitative study aimed to assess the factors influencing consumers’ choices regarding food labeling models to identify products displayed at food services. The foods chosen for this study were three different sandwiches.

Data were collected from April to May 2020. Adults aged 18 and older who accepted participating in the study and lived in the Rio Grande do Sul (RS) State (Brazil) were included. The exclusion criteria included those who were not habitual sandwich consumers since this was selected for this comparative analysis. In addition, subjects that did not answer the entire questionnaire were also excluded from the database.

The sample size considered the population focus of this research (adults aged 18 and older living in RS State, Brazil). As the RS state has 8,274,860 inhabitants, at a 95% confidence level, the calculated study sample was 385 individuals (SurveyMonkey Audience^®^, San Mateo, CA, USA).

### 2.2. Survey Procedure

Data collection was performed via an online questionnaire using the SurveyMonkey^®^ platform. Subjects were authorized to fill out the questionnaire after giving informed consent to participate in the research. Twenty-six questions comprised the form, divided into three sections: (1) sociodemographic questions; (2) evaluation of sandwich choice and intervening aspects; and (3) food label understanding and perceptions.

(1) Sociodemographic questions:

We asked six closed-ended questions to characterize the profiles of the respondents. The questions were about: gender, age, municipality of residence, schooling, and health condition or lifestyle requiring the restriction of some food, nutrient, or ingredient. For the subjects who answered positively to the last question, we asked them to indicate these conditions. Respondents could mark more than one condition.

**Figure 2:**
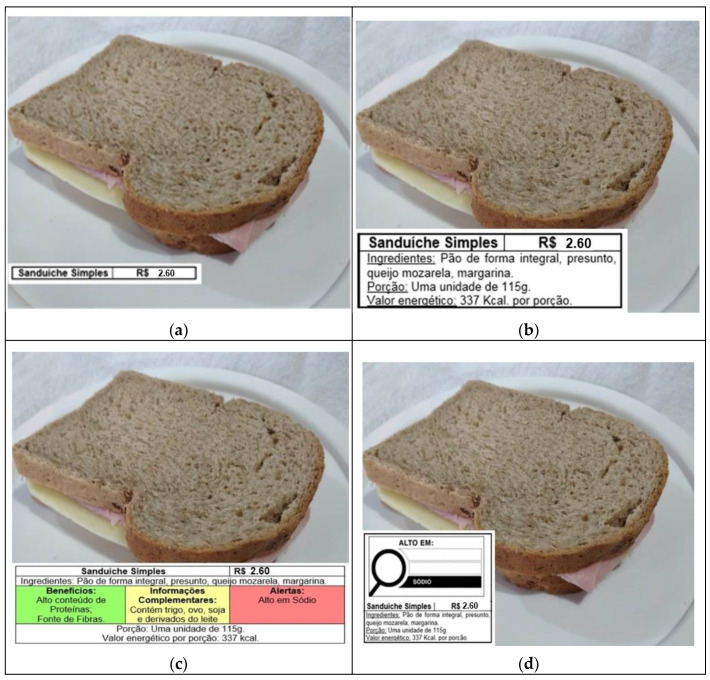
Food labeling models placed on the simple sandwich, Brazil, 2020. (**a**) Traditional label; (**b**) Simplified label; (**c**) Traffic-light label; (**d**) Warning label.

**Figure 3:**
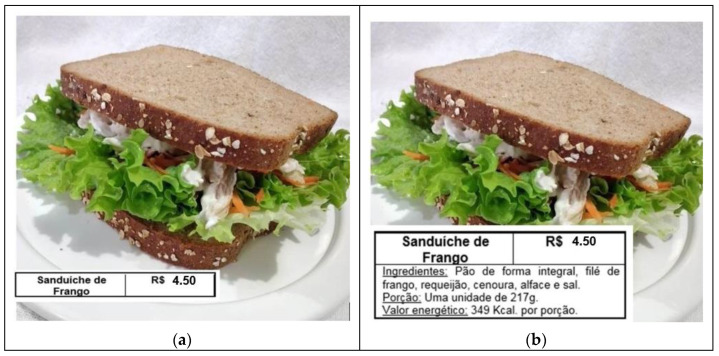

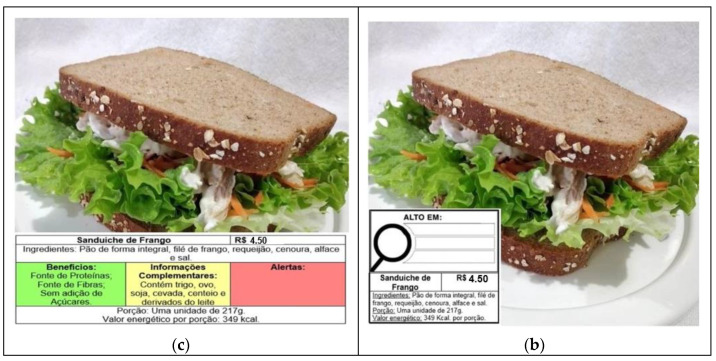
Food labeling models placed on the chicken sandwich, Brazil, 2020. (**a**) Traditional label; (**b**) Simplified label; (**c**) Traffic-light label; (**d**) Warning label.

**Figure 4:**
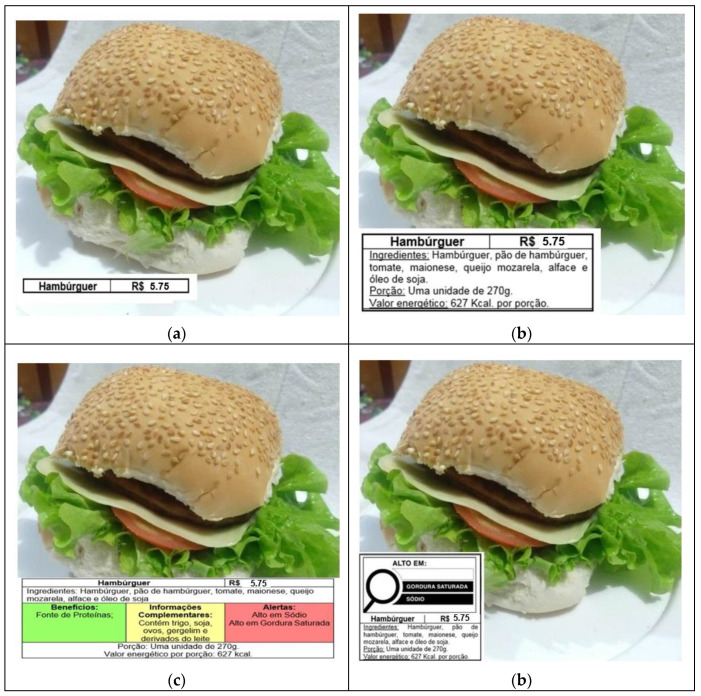
Food labeling models placed on the hamburger, Brazil, 2020. (**a**) Traditional label; (**b**) Simplified label; (**c**) Traffic-light label; (**d**) Warning label.

(2) Evaluation of sandwich choice and intervening aspects:

The respondents evaluated three types of sandwiches presented with four different format labels (traditional, simplified, traffic-light, and warning) (Figure 2). The “simple sandwich” ingredients were whole wheat bread, ham, cheese, and margarine (Figure 2). The “chicken sandwich” contained whole wheat bread, chicken breast, cream cheese, carrot, lettuce, and salt (Figure 3). Lastly, the “hamburger” ingredients were hamburger bun, beef patty, tomato, mayonnaise, cheese, lettuce, and soybean oil (Figure 4).

English translations: (a) Simple Sandwich. Price in Brazilian currency. (b) Simple Sandwich. Price in Brazilian currency. Ingredients: whole wheat bread, ham, mozzarella cheese, margarine. Portion: one unit of 115 g. Calories per serving: 337 Kcal. (c) Simple Sandwich. Price in Brazilian currency. Ingredients: whole wheat bread, ham, mozzarella cheese, margarine. Benefits (in green): High in proteins; Source of fibers. Complementary information (in amber): Contains wheat, egg, soy, and milk derivatives. Warnings (in red): High in sodium. Portion: one unit of 115 g. Calories per serving: 337 Kcal. (d) High in (magnifying glass): sodium. Simple Sandwich. Price in Brazilian currency. Ingredients: whole wheat bread, ham, mozzarella cheese, margarine. Portion: one unit of 115 g. Calories per serving: 337 Kcal.

The three types of sandwich pictures were presented to the subjects four times. Each time the label was different, and the subjects were asked to click on the sandwich of their preference. After each presentation, the subjects answered questions about the central aspect influencing their choices. The options were: price, taste preference, appearance, nutritional quality, do not know, and other (white space for specification).

English translations: (a) Chicken Sandwich. Price in Brazilian currency. (b) Chicken Sandwich. Price in Brazilian currency. Ingredients: whole wheat bread, chicken breast, cream cheese, carrot, lettuce, and salt. Portion: one unit of 217 g. Calories per serving: 349 Kcal. (c) Chicken Sandwich. Price in Brazilian currency. Ingredients: whole wheat bread, chicken breast, cream cheese, carrot, lettuce, and salt. Benefits (in green): High in proteins; Source of fibers; No added sugar. Complementary information (in amber): Contains wheat, egg, soy, barley, rye, and milk derivatives. Warnings (in red): none. Portion: one unit of 217 g. Calories per serving: 349 Kcal. (d) High in (magnifying glass): none. Chicken Sandwich. Price in Brazilian currency. Ingredients: whole wheat bread, chicken breast, cream cheese, carrot, lettuce, and salt. Portion: one unit of 217 g. Calories per serving: 349 Kcal.

The first label presented was the traditional model, which included the name of the preparation and the price (Figure 2a, Figure 3a and Figure 4a). The second was the simplified format. Besides the information conveyed on the traditional model, the simplified model also included the list of ingredients used to prepare the product (in descending order by amount), serving size, and energy value (Figure 2b, Figure 3b and Figure 4b).

The traffic-light system was the third one. It portrayed all the nutritional information shown in the simplified model and a panel with stoplight colors: green, amber, and red. The green light highlighted some benefits of the preparation, the amber light listed complementary information on the presence of allergens [14], and the red light provided health warnings [12] (Figure 2c, Figure 3c and Figure 4c).

English translations: (a) Hamburger. Price in Brazilian currency. (b) Hamburger. Price in Brazilian currency. Ingredients: beef patty, hamburger bun, tomato, mayonnaise, mozzarella cheese, lettuce, and soybean oil. Portion: one unit of 270 g. Calories per serving: 627 Kcal. (c) Hamburger. Price in Brazilian currency. Ingredients: beef patty, hamburger bun, tomato, mayonnaise, mozzarella cheese, lettuce, and soybean oil. Benefits (in green): Source of proteins. Complementary information (in amber): Contains wheat, soy, eggs, sesame seeds, and milk derivatives. Warnings (in red): High in sodium; High in saturated fat. Portion: one unit of 270 g. Calories per serving: 627 Kcal. (d) High in (magnifying glass): saturated fat; sodium.

Finally, the warning format used the same information indicated in the simplified model and showed a magnifying glass symbol (Figure 2d, Figure 3d and Figure 4d). Again, we used the ANVISA’s nutritional recommendations for a front-of-package label of industrialized products as a model. The magnifying glass highlighted a ‘High in’ warning indicating high levels of added sugar, saturated fat, and sodium [12].

(3) Food label—preference, understanding, and perceptions:

Based on the hypothesis that the type of label influences the choice of food, a preference ranking test was applied to determine the subjects’ preference towards the proposed food labeling models. The assigned score ranged from 1 to 4, the most and the least preferred, respectively. The subjects also indicated their level of understanding of the food labeling models. After each model was shown separately, they marked their level of understanding among the options: excellent, good, regular, bad, and poor.

To measure acceptability, we asked the question: “how much would you like to find this label on all foods at a snack bar?”. Consumer acceptability was scored on a 5-point hedonic scale from “like very much” to “dislike very much”. The statistical treatment considered both extremes: “like very much” with “like slightly” and “dislike very much” with “dislike slightly”.

The questionnaire included three general statements to verify: (1) if consumers believe the information provided on food items at food services is enough; (2) if it would be necessary to complement such information; and (3) the need for legislation to regulate food labeling at food services. In addition, the respondents had to answer a 5-point Likert scale to specify their level of agreement to the statements (from “strongly agree” to “strongly disagree”). Lastly, the subjects could express themselves freely in an open-ended question, making comments and leaving suggestions about the nutrition labeling models for food services.

### 2.3. Data Analysis

We created an Excel data table with the results for subsequent analyses by SPSS (Version 25.0). We applied Pearson’s chi-square test for the nominal variables and the Friedman test for the ordinal data with a 0.05 level of significance. The Friedman tests were used to identify differences between consumers’ preference for a given label and their choices. The open-ended questions were evaluated by a qualitative analysis and a search for explanations and discussion of the quantitative findings.

### 2.4. Ethics Committee

This study was approved by the Research Ethics Committee at Universidade Franciscana (UFN) and is registered under Protocol No. 1.877.129. It is part of a project called Elaboração de software para desenvolvimento de informativos nutricionais para serviços de alimentação—Elaboration of software for development of nutrition labels for food services, loosely translated; an amendment was prepared to request the extension of the project period. The research was performed in accordance with the ethical standards as laid down in Resolution 466/2012 [28].

## 3. Results

### 3.1. Subject Profiles (Sociodemographic Questions)

A total of 459 subjects initiated the survey. Nevertheless, 9 subjects were excluded for not living in RS State (Brazil), 32 for not being habitual sandwich consumers, and 5 for not answering the entire questionnaire. Therefore, the final sample included 413 subjects. The average age of the respondents was 35.5 (SD: 14.4) years; the other sociodemographic information can be found in Table 1.

Most subjects (80.4%; *n* = 332) indicated that they did not have a health condition or lifestyle that required restriction of some food, nutrient, or ingredient (Table 2). Of those with dietary restrictions, 40.7% reported to be “lactose intolerant”; 25.9% (*n* = 21) chose the option “healthy eating”, and 14.8% (*n* = 12) answered “vegetarianism”. The subjects left in the white space the following comments (one subject per comment): veganism, high triglycerides and cholesterol levels, gastritis, Crohn’s disease, hypoglycemia, hyperinsulinemia, atopic dermatitis, fibromyalgia, reflux, ulcerative rectocolitis, hypothyroidism, and low carb diet.

### 3.2. Sandwich Choice and Intervening Aspects

The sandwiches chosen according to the labeling model are in Table 3. It is noteworthy that the chicken sandwich was the first option regardless of the food label. However, better choices were related to increased information presented to consumers.

The content of Table 2 shows an association between the reason for the choice of sandwich and the food label model (*p* < 0.001). An analysis of the nutritional quality criterion indicated higher percentages for the traffic-light (72.7%; *n* = 293) and warning (71.8%; *n* = 285) models than for the simplified (50%; *n* = 200) and traditional (32.3%; *n* = 127) formats.

### 3.3. Food Label—Preference, Understanding, and Perceptions

There was an association between the level of preference and the kind of food label; the traffic-light label (97.4%; *n* = 402) had the most excellent acceptability, followed by the warning label (93.4%; *n* = 386) and the simplified (92.5%; *n* = 382) models (*p* = 0.003). In general, all four models had good acceptability (Table 2).

The preference test indicated statistically significant differences regarding the types of food labels (*p* < 0.001). The outcomes revealed differences for all two-by-two comparisons of models (*p* < 0.001), except between the simplified and warning labels (*p* = 0.122). The traffic-light label was the preferred model (Table 2).

#### 3.3.1. Consumers’ Understanding about the Food Labeling Models

The traffic-light and warning models had a better level of understanding. Nevertheless, the understanding of the simplified label was considered good (Table 2). The level of understanding and the kind of food label showed an association between the parameters. There was a higher level of understanding of the traffic-light label (70.5%, *n* = 291) than of the warning (49.2%, *n* = 203) and simplified (32.4%, *n* = 134) formats (*p* < 0.001).

#### 3.3.2. General Opinion on Nutrition Labels at Food Services

In regards to the statement saying that the nutritional information currently provided at snack bars and restaurants is sufficient, 70% (*n* = 329) replied they “disagree” or “strongly disagree”. However, most of the consumers (96.1%; *n* = 397) replied they “agree” or “strongly agree” that it is necessary to complement the information placed on foods with ingredients and the number of calories per serving. Furthermore, 94.5% (*n* = 389) of the respondents agreed that it is essential to have legislation regulating nutrition labels accompanying food items on display at snack bars and buffets at restaurants (Table 4).

Thus, the traffic-light and warning nutrition labeling models led to a reduction in the choice of the simple sandwich and the hamburger. This outcome was corroborated by the answers (statements below) obtained in the open-ended questions:

“I agreed with all labels and believed they could totally influence people to improve their eating habits.”

(line 70)

“The commented items will really help when choosing food for consumption.”

(line 250)

“We are more aware of food labels. I think it is important to clarify.”

(line 204)

The subjects also manifested themselves spontaneously concerning the information in the alert model (statements below):

“Too much sodium”

(line 310)

“Without any warning”

(line 70)

“Does not have a warning”

(line 70)

“Does not have warnings”

(line 386)

“Has the information that it does not have any warning”

(line 288)

Surprisingly, uncertainty about the sanitary aspects of the salads and the sandwiches was also addressed (statements below):

“Worry about the washing of greens and vegetables”

(line 377)

“For not eating green salad away from home, due to uncertainty about how it was washed (or if it was washed)”

(line 231)

“I am afraid of eating uncooked salad where I am not sure it was properly washed”

(line 279)

## 4. Discussion

### 4.1. Subject Profiles

The sociodemographic profile of the sample was mainly composed of young adult women with a high level of education. Some of these characteristics are predictors of healthier lifestyles and a higher level of nutrition literacy (NL). A study [29] addressed NL as skills that enable the subjects to access nutrition information and perform screening according to its quality. Sequentially, these subjects can transform this acquired understanding into positive aspects for their diet. This possible association among the characteristics of the analyzed subjects and a high NL is interesting, as it may also be related to an adequate ability to read food labels [29].

Although lactose intolerance was the most mentioned dietary restriction (40.7%), a similar percentage of the sample (40.7%) referred to restrictions defined in terms of healthier lifestyles (“healthy eating” and “vegetarianism”). If we include calorie-restricted diets for weight loss, this percentage rises to 50.5%. These data may corroborate the idea that the subjects have a moderate NL and, for this reason, have a reasonable understanding of the content of food labels [30].

The use of the internet to carry out scientific research may have contributed to the subjects’ characteristics. The internet has been recommended for its advantages, mainly related to its low cost, breadth, and speed. Moreover, considering the moment of the COVID-19 pandemic, when this research was carried out, the tool proved to be even more opportune due to the social isolation required. However, there is a trend towards greater participation by specific groups. Usually, the majority participation of women and young people with a high socioeconomic and educational levels, is observed, as the population sample of this research was described [31,32].

### 4.2. Sandwich Choice and Intervening Aspects

**Figure 5:**
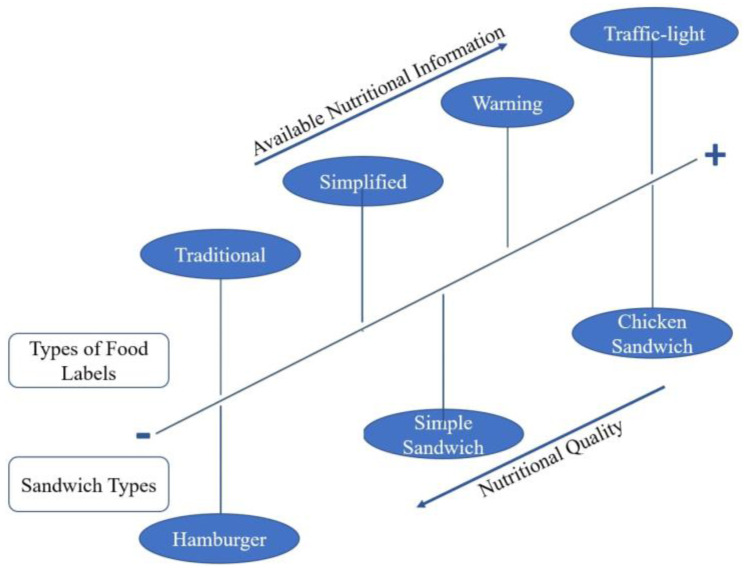
Level of information in the nutrition labeling models and the nutritional quality of the sandwiches used to perform the current study.

The food labeling models tested in this study present distinct levels of information, and there is also a variation in the nutritional quality of the sandwiches (Figure 2). The criteria depicted in Figure 5 were followed to assess the choices made by consumers regarding the different nutrition labels on the sandwiches; the chicken sandwich was considered to have the highest nutritional quality, and the traffic-light was the model conveying more information.

In addition to Brazil, other countries are also studying how food labels can interfere with food choices. For example, in Germany, 420 adults underwent a randomized experimental study. Four label models were tested, in addition to the non-presentation of nutritional information. The traffic-light model proved to be the easiest to understand for this population. However, the study highlights that the label alone cannot change consumer attitudes and practices [26].

A different example is “The New Zealand Starlight Study” [33]. The research consisted of a four-week randomized, controlled trial that investigated the effects of nutrition labels on consumer food purchases. According to the authors, during the research, the participants (*n* = 1255) could consult on their smartphones the nutrition labels of 66,915 packaged products using the barcode. The results showed a decreasing interest in the consultation over the period. Another important aspect was the greater interest in labels for certain types of food, such as cereals and snacks, and less interest in foods with a lower level of processing, such as eggs and meat. The results also indicated that people generally obtain healthier products when the consultation is carried out immediately before the purchase. The outcomes reinforce the importance of the information present on the packaging. They also highlight the need to facilitate the consultation of this information through different models and devices [33].

The subjects were able to indicate the reason for their choice after the presentation of the products and the different models of labels with their information (Table 2). The price is not among the leading factors influencing the food choice (<2.5%). Nutritional quality and taste preference were the major determinant factors (Table 2). The outcomes were not surprising, considering the profile of the investigated subjects. As mentioned, nutritional information on the label is not necessarily a predictor of healthier choices [26]. The characteristics observed in the studied population may have been one of the causes for attention in this sense.

According to Table 3, the consumer’s choice regarding the nutritional quality is proportional to the information presented in the products offered for sale. The choice of sandwich related to nutritional quality was significantly higher in the traffic-light and warning models than in the others. The strategy of using front-of-pack nutrition labels is aimed at helping people make healthier food choices, which ultimately helps control the growing number of obese and overweight people [34].

The results point to a behavior trend according to the type of label presented. Even with many variables interfering with the choice, such as flavor, ingredients, etc. [35], we observed a pattern of behavior. For all sandwiches presented, the greater amount of information revealing the nutritional quality of the product, possibly, allowed a healthier choice.

### 4.3. Food Label—Preference, Understanding, and Perceptions

As for consumers’ acceptability regarding the types of food labels, all proposed models were well accepted. This result indicates that consumers approve of having more information when consuming meals and snacks in food services. Studies have shown that the more information the consumer obtains when buying a product/food, the better it is for their health and, consequently, their quality of life [27,34,36,37,38].

As in the German study [26], the traffic-light model was preferred, possibly due to providing more information (Figure 5). Traffic-light nutrition labels offer plenty of information. They use a panel with stoplight colors: the green light indicates possible benefits; the amber light indicates complementary tips on the presence of allergens; and the red light indicates health warnings. Another study evaluated the efficacy of three labeling formats: nutritional table, guideline daily amounts, and traffic-light. The researchers observed that the amount of information communicated by each model influenced the subjects’ choices, and the traffic-light format was better accepted [27].

However, the traffic-light labeling model may contain too much information, and the sandwich consumers may not have observed this on the online form. Therefore, the best way to conduct this kind of evaluation is to perform an in loco test at food service locations, presenting several foods with the same label; only then is it possible to determine consumers’ understanding.

Anyway, good purchase intentions are associated with information on food packaging. There is evidence of positive results regardless of the model used to convey the information. A study compared nutrition claims, which contain only textual information, with information system by-products (guarantee seals) and had better success with the first model. In this case, the products with packages containing only textual information were more likely to be purchased. Therefore, variations among the target population must be considered to adopt the best strategy [37,38].

The critical point must be understanding the transmitted message and transforming it into healthy behavior. Thus, when we asked the subjects whether the messages on the labels were understandable, the traffic-light had the highest percentage of comprehension (70.5%, *n* = 291). To better understand the reason for this result, they manifested their opinions (statements below):

“It would be good if all foods had a traffic-light label, which would call more attention for the people to consume”

(line 97)

“I liked the traffic-light label because even if the consumer does not fully understand the ingredients, at least he/she will have the color signals. I think the model with a magnifying glass is good, though inefficient because if the consumer does not understand what “saturated fat” is, he/she will not be able to say if it is good or not to have a high level of this in the food. The traditional model is not so good, since it does not indicate the benefits or disadvantages”

(line 637)

“The traffic-light model is more pleasant because it shows both sides of the food, not dichotomizing into “unhealthy” and “healthy”

(line 177)

“The warnings are important, but I think the traffic-light model is very good, with useful and accessible information”

(line 159)

The current results agree with the data reported by the authors of [27]. The authors concluded that the traffic-light model requires less time to understand and can affect purchasing decisions. However, it is unlikely that the food label alone can change the perception of a particular food.

On the other hand, the warnings had a more significant effect on parents’ health perception than the traffic-light system. Even though these findings indicate that using labels, e.g., warning logos, in industrialized products may lower the health perception of items having an unfavorable nutrient profile [34].

Therefore, consumers tend to make better food choices when nutritional labels communicate more information, as is the case of the traffic-light model. The more specific and concise the information, the greater the chances of making inappropriate food choices [34]. Conversely, some authors claim that models with a lot of information and colors may distract consumers, who end up not paying attention to the nutrition facts stated on the labels [38].

Familiarity with nutrition labeling schemes directly affects the choice of products. Because of that, understanding of such models may be a predictor for future consumers’ assessments. Another consideration is that the comprehension assessments were self-reported in the present study. This aspect is a limitation that can be remedied in future studies by applying, for example, the nutrition literacy assessment instrument, specifically the part on food labels [39].

The instrument used also assessed whether the subjects considered the information provided at the attended services to be sufficient. In this sense, they believe there is a lack of data on the nutritional composition of the products displayed at snack bars and restaurants. They also consider that, when available, the labels are not sufficient to clarify the nutritional quality. Moreover, they agree that regulating the nutritional information accompanying foods on display at snack bars and buffets at restaurants is essential. Such data support the relevance of conducting studies in this area. Furthermore, this research should soon be implemented into practice so that society can use their benefits.

## 5. Conclusions

Nutrition labels are an essential tool to guide consumers about the quality and quantity of the nutritional constituents of foods. They allow the consumer to make purchase decisions based on the information provided. Among the formats of food labels explored, the traffic light was the best accepted and understood. Based on the information provided, consumers were engaged in managing their health and were seeking healthier food options.

Within the studied context, we observed that the presence of food labels in food services is a consumer demand. The outcomes reveal important implications for future educational or policy campaigns. In addition, the use of this tool for food service owners and managers can be a great competitive opportunity. The standardization of food labeling requires greater formalization and control of processes. The implementation of food labels can, thus, become an advantage.

The use of nutrition labels is well accepted by consumers and can contribute to increasing their understanding. However, the advantages seem to be limited to consumer participation in the structuring of the tool. On the other hand, efforts are needed to make consumers aware of the importance of reading labels. Educational actions in this sense can help to make better choices based on an adequate understanding of the information attached to the product. If we add up all the aspects mentioned, the positive social and economic impact may outweigh the initial investments. The population is improved by better food choices and the resulting health benefits. The government may reduce investments in hospitalizations and other assistance actions. Finally, food services will be able to improve the management of their processes, in line with the general interests of the population.

### Study Limitations

The application of an online questionnaire replacing an on-site assessment at the food service was an important limitation. Food choice is multifactorial. Therefore, the experience of purchasing food is linked to several aspects that are not reproduced virtually. The product layout, font size, packaging, and availability of the subject at the time of purchase are examples that can influence the results obtained in a face-to-face scenario. Therefore, the continuity of the research in different states of Brazil may clarify some important aspects. We highlight that Brazil has a high territorial dimension with great population diversity. Therefore, the results can be compared to other world regions, as long as different audiences and realities are considered.

## Figures and Tables

**Table 1:** Sociodemographic characteristics of the participants.

Variables	*n* = 413	%
Gender		
Female	355	86.0
Male	58	14.00
Educational level		
Complete High School	45	11.0
Incomplete University Education	122	29.5
Complete University Education	115	27.8
Master’s and/or PhD	131	31.7

**Table 2:** Dietary restrictions and care reported by consumers, Rio Grande do Sul, Brazil.

Dietary Restrictions and Care	*n*	%
Lactose intolerance	33	40.7
Healthy eating	21	25.9
Vegetarianism	12	14.8
Diabetes Mellitus	9	11.1
Obesity/Overweight	8	9.8
Irritable bowel syndrome	8	9.8
Systemic Arterial Hypertension (SAH)	7	8.6
Celiac disease	5	6.2
Food allergy	2	2.5

**Table 3:** Influence of different food labels on the choice of sandwiches by consumers, Rio Grande do Sul, Brazil.

	Traditional *n* (%)	Simplified *n* (%)	Warning *n* (%)	Traffic-Light *n* (%)
**Influence of the food labeling models on the choice of sandwich by consumers**				
Simple Sandwich	31 (7.5)	31 (7.5)	19 (4.6)	22 (5.3)
Chicken Sadwich	3030 (73.4)	331 (80.2)	362 (87.7)	358 (86.7)
Hamburger	79 (19.1)	51 (12.3)	32 (7.7)	33 (8.0)
Reason for the choice *				
Nutritional quality	127 (32.3)	200 (50.0)	285 (71.8)	293 (72.7)
Taste preference	143 (36.4)	143 (35.8)	82 (20.7)	89 (22.1)
Appearance	114 (29.0)	47 (11.8)	27 (6.8)	21 (5.1)
Price	9 (2.3)	10 (2.5)	3 (0.8)	0 (0.0)
Food labeling model preference **				
Like very much	-	275 (66.6)	250 (60.5)	317 (76.8)
Like slighfly	-	107 (25.9)	136 (32.9)	85 (20.6)
Neither like nor dislike	-	27 (6.5)	17 (4.1)	8 (1.9)
Dislike slighfly	-	3 (0.7)	7 (1.7)	2 (0.2)
Deslike very much	-	1 (0.2)	2 (0.5)	0 (0.0)
Preference test ***				
	1374 ^a,b,c^	1089 ^a,d^	1002 ^c,e^	755 ^b,d,e^
Consumer understanding level *				
Great		134 (32.4)	291 (70.5)	203 (49.2)
Good		154 (37.3)	94 (22.8)	160 (38.7)
Regular		89 (21.5)	17 (4.1)	29 (7.0)
Bad		31 (7.5)	6 (1.5)	12 (2.9)
Poor		5 (1.2)	4 (1.0)	8 (1.9)

* Pearson’s chi-square test (*p* < 0.001). ** Pearson´s chi-square test (*p* = 0.003). *** Similar letters indicate a significant difference (*p* < 0.001).

**Table 4:** Opinion and understanding of sandwich consumers about nutrition labels, Rio Grande do Sul, Brazil.

Question	Completely Agree	Agree	Indifferent	Disagree	Completely Disagree
- Currently, the present information about the foods displayed for sale in snack bars and restaurant buffets is sufficient.	18 (4.5)	36 (9.0)	25 (6.5)	247 (60.0)	82 (20.0)
- It is necessary to complement the present information about the food with ingredients, caloric value in the portion, among other aspects.	261 (63.2)	136 (32.9)	10 (2.4)	1 (0.2)	0 (0.0)
- It is essential to have legislation to regulate the nutritional information on foods displayed in snack bars and restaurant buffets.	256 (62.0)	133 (32.5)	16 (4.0)	2 (0.5)	1 (0.2)

## Data Availability

The data are is contained within the article.
